# Performance of Retrieval-Augmented Generation Large Language Models in Guideline-Concordant Prostate-Specific Antigen Testing: Comparative Study With Junior Clinicians

**DOI:** 10.2196/78393

**Published:** 2025-11-19

**Authors:** Joshua Yi Min Tung, Quan Le, Jinxuan Yao, Yifei Huang, Daniel Yan Zheng Lim, Gerald Gui Ren Sng, Rachel Shu En Lau, Yu Guang Tan, Kenneth Chen, Kae Jack Tay, Jen Hong Tan, John Shyi Peng Yuen, Christopher Wai Sam Cheng, Henry Sun Sien Ho

**Affiliations:** 1 Data Science and Artificial Intelligence Laboratory, Singapore General Hospital, Singapore, Singapore; 2 Department of Urology, Singapore General Hospital, Block 4 Level 1, 16 College Road, Singapore, 169854, Singapore, 65 62223322; 3 Department of Gastroenterology, Singapore General Hospital, Singapore, Singapore; 4 Department of Endocrinology, Singapore General Hospital, Singapore, Singapore

**Keywords:** artificial intelligence, AI, large language model, LLM, guideline concordance, junior clinician

## Abstract

**Background:**

Prostate-specific antigen (PSA) testing remains the cornerstone of early prostate cancer detection. Society guidelines for prostate cancer screening via PSA testing serve to standardize patient care and are often used by trainees, junior staff, or generalist medical practitioners to guide medical decision-making. However, adherence to guidelines is a time-consuming and challenging task, and rates of inappropriate PSA testing are high. Retrieval-augmented generation (RAG) is a method to enhance the reliability of large language models (LLMs) by grounding responses in trusted external sources.

**Objective:**

This study aimed to evaluate a RAG-enhanced LLM system, grounded in current European Association of Urology and American Urological Association guidelines, to assess its effectiveness in providing guideline-concordant PSA screening recommendations compared to junior clinicians.

**Methods:**

A series of 44 fictional outpatient case scenarios was developed to represent a broad spectrum of clinical presentations. A RAG pipeline was developed, comprising a life expectancy estimation module based on the Charlson Comorbidity Index, followed by LLM-generated recommendations constrained to retrieved excerpts from the European Association of Urology and American Urological Association guidelines. Five junior clinicians were tasked to provide PSA testing recommendations for the same scenarios in closed-book and open-book formats. Answers were compared for accuracy in a binomial fashion. Fleiss κ was computed to assess interrater agreement among clinicians.

**Results:**

The RAG-LLM tool provided guideline-concordant recommendations in 95.5% (210/220) of case scenarios, compared to junior clinicians, who were correct in 62.3% (137/220) of scenarios in a closed-book format and 74.1% (163/220) of scenarios in an open-book format. The difference was statistically significant for both closed-book (*P*<.001) and open-book (*P*<.001) formats. Interrater agreement among clinicians was fair, with Fleiss κ of 0.294 and 0.321 for closed-book and open-book formats, respectively.

**Conclusions:**

Use of RAG techniques allows LLMs to integrate complex guidelines into day-to-day medical decision-making. RAG-LLM tools in urology have the capability to enhance clinical decision-making by providing guideline-concordant recommendations for PSA testing, potentially improving the consistency of health care delivery, reducing cognitive load on clinicians, and reducing unnecessary investigations and costs. While this study used synthetic cases in a controlled simulation environment, it establishes a foundation for future validation in real-world clinical settings.

## Introduction

Prostate cancer is the second most commonly diagnosed cancer and the fifth leading cause of cancer-related death among men globally [1]. Screening for prostate cancer is thus a common issue in both primary and specialist care settings. Prostate-specific antigen (PSA) testing is the most widely used method for early detection, but remains a controversial issue in urological literature, largely owing to the harms associated with overdiagnosis and overtreatment [2,3].

Society guidelines for prostate cancer screening via PSA testing serve to streamline and standardize patient care and are often used by trainees, junior staff, or nonspecialist medical practitioners to guide medical decision-making. Such guidelines have been issued by various organizations such as the European Association of Urology (EAU) [4] and American Urological Association (AUA) [5], but discrepancies between these guidelines, such as recommendations on whether PSA screening should be offered, the appropriate patient populations, and screening intervals, pose challenges for clinical decision-making. These are further complicated by the need to consider other patient factors, such as the need to calculate estimated life expectancy (as many guidelines do not recommend PSA screening in patients with a <10- or <15-year life expectancy), and the need to consider the patient’s own preferences. Shared decision-making forms a key component in both the EAU and AUA guidelines, particularly in older men or those with multiple medical comorbidities.

The current EAU-European Association of Nuclear Medicine-European Society for Radiotherapy and Oncology-European Society of Urogenital Radiology-International Society of Urological Pathology-International Society of Geriatric Oncology and AUA and Society of Urologic Oncology guidelines on prostate cancer and early detection of prostate cancer stand at 239 and 47 pages, respectively. Appropriate decision-making and adherence to guidelines is therefore a time-consuming and challenging task for nonspecialists in a primary care setting, as well as for specialists in outpatient settings where time constraints are common. Prior studies have shown a low rate of compliance to organizational guidelines, such as a cohort study of 32,306 men showing that 40% of those aged >80 years received inappropriate PSA screening [6].

One potential solution to this problem is to use artificial intelligence (AI) to parse guidelines and deliver an appropriate recommendation. Large language models (LLMs) are a form of AI that are trained on large amounts of text data and hence have the capability to process unstructured text inputs and generate appropriate responses. They can thus be applied in health care, such as in patient communications, education, and clinical risk stratification [7]. However, general LLMs, such as the GPT models developed by OpenAI, are not specifically designed for health care use and can produce inaccurate or misleading information. They have a knowledge cutoff based on the recency of the underlying training data, for example, January 2022 for the OpenAI GPT-4 models. To address these limitations, retrieval-augmented generation (RAG) techniques have been developed to enhance the accuracy of LLMs. RAG directs the LLM to answer a given scenario by referencing an additional database of curated information, such as a set of guidelines. By grounding the responses using relevant information from the database, LLMs can overcome their intrinsic knowledge cutoff and produce responses with less hallucination [8-10].

Thus, the aim of this study was to evaluate the accuracy of a RAG-enabled LLM that had been grounded in the EAU and AUA guidelines pertaining to prostate cancer screening.

## Methods

### Ethical Considerations

This study was conducted in a simulated environment using only fictional patient data. As the use of fictional data does not fall under local Human Biomedical Research Act regulations, ethics approval was not required.

### Development of Case Scenarios

A series of 44 fictional case scenarios was developed to reflect a range of clinical presentations at an outpatient clinic setting. These free-text scenarios included fictional patient biodata such as age, medical comorbidities, presence or absence of urological symptoms (eg, hematuria or lower urinary tract symptoms, if any), and prior PSA readings (if applicable). These were written by a urology fellow with 8 years of clinical experience and supervised by 2 urology consultants with >20 years of clinical experience each.

### Development of the RAG-Enabled LLM

**Figure 1.**
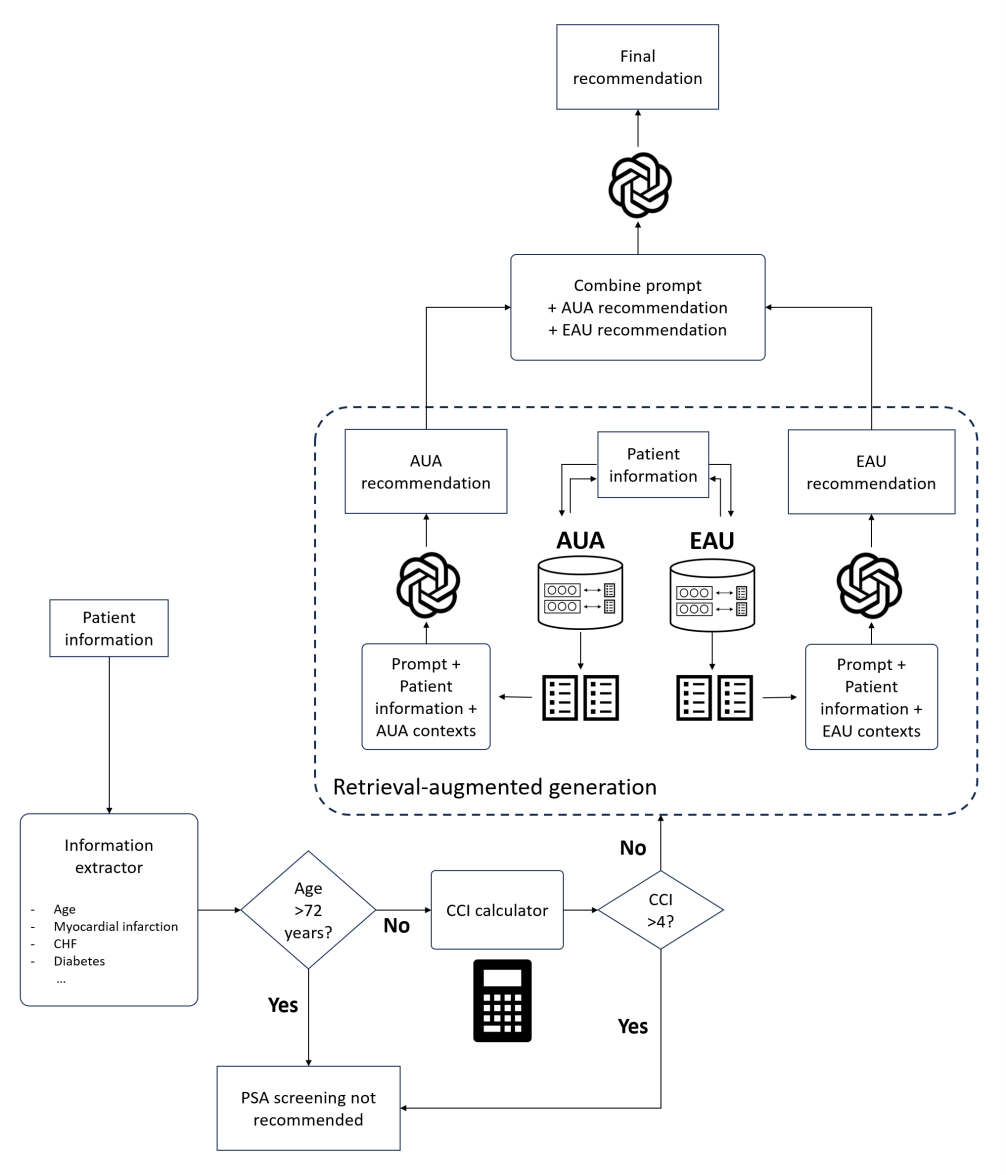
Workflow schematic of the retrieval-augmented generation–enabled large language model pipeline for prostate-specific antigen (PSA) testing recommendations. AUA: American Urological Association; CCI: Charlson Comorbidity Index; CHF: congestive heart failure; EAU: European Association of Urology.

We developed an automated pipeline to process case scenarios based on how a health care provider would provide a PSA testing recommendation. The schematic diagram is shown in Figure 1.

Key components of this pipeline included an LLM-based calculator to extract relevant patient information (age and comorbidities) from the case scenario, to calculate the Charlson Comorbidity Index (CCI) and thereby estimate the expected 10-year life expectancy. Patients who were not expected to live at least 10 years were not recommended for PSA screening [4,5], and the pipeline did not allow such case scenarios to proceed. Likewise, scenarios where the patient was aged >72 years were also not permitted to proceed. We provide further technical details of the CCI calculator in Multimedia Appendix 1 [3-5,11,12].

For patients with at least a 10-year life expectancy based on CCI scores, a RAG-enabled LLM was used to provide a recommendation based on the given case scenario. In comparison with standard “off-the-shelf” LLMs that are not trained on domain-specific medical information, RAG allows the LLM to reference a fixed set of material, such as the relevant EAU and AUA society guidelines in this study. Language models augmented in this way with contextualized information can overcome their intrinsic knowledge deficits and reduce hallucination by constraining their responses to the provided information.

Because the AUA and EAU guidelines occasionally provide different and nonoverlapping recommendations, separate answers were first generated from each set of guidelines and then combined to produce the final recommendations.

We provide further technical details of the RAG-enabled LLM in Multimedia Appendix 1 [3-5,11,12]. These include explanations of modern RAG techniques applied to optimize performance, such as context filtering to improve retrieval of relevant information and advanced prompting methods (chain-of-thought reasoning [13], constraining responses to retrieved information, providing example output structures, and using an expert clinician persona). The full RAG prompt can be found in Multimedia Appendix 1 [3-5,11,12].

### Relevant Software

The RAG prototype was developed with Python (version 3.10; Python Software Foundation). Vector databases were constructed using Unstructured API for ingestion of PDF documents, OpenAI API for generation of text embeddings, and Qdrant as the vector database. For LLM calls, we used both OpenAI and Anthropic APIs for different components in our pipeline. We used both LlamaIndex and Langchain for orchestration, with LlamaIndex handling retrieval of augmented generation components, whereas Langchain was used for structured data extraction and connecting pipeline components.

### Answer Generation and Grading

Five junior clinicians were tasked to provide recommendations on PSA testing for each of the case scenarios. They included a first-year medical officer, a second-year family medicine resident, 2 second-year urology residents, and a third-year urology resident. Each clinician completed the task in a “closed-book” format, followed by an “open-book” format in which they were permitted to reference relevant material of their choice (eg, guidelines or textbooks). The time taken to complete the task in each format was recorded.

The RAG-LLM tool was likewise provided with the same set of fictional case scenarios and instructed to provide recommendations on PSA testing. We conducted 5 runs to assess the consistency of the LLM output. Answers were graded by the study team in a binomial format (correct or incorrect). Answers were marked as correct if they were concordant with either the EAU or AUA guidelines.

### Statistical Analysis

SPSS (version 26.0; IBM Corp) was used for the statistical analysis of quantitative data. Answers from the RAG-LLM tool and human comparators were compared using Student 2-tailed *t* test. Interrater agreement was calculated using Fleiss κ.

## Results

The RAG-LLM tool provided guideline-concordant recommendations in 95.5% (210/220) of case scenarios, compared to junior clinicians, who were correct in 62.3% (137/220) of scenarios in a closed-book format and 74.1% (163/220) of scenarios in an open-book format. The difference was statistically significant for both closed-book (*P*<.001) and open-book (*P*<.001) formats.

Cases were divided into screening (20/44, 45.5%) and follow-up (24/44, 54.5%) categories. The RAG-LLM tool provided an incorrect recommendation in 1 screening case: in all 5 instances, it failed to recommend a PSA test for a patient for whom screening was recommended. In comparison, junior clinicians missed 16/100 (16%) tests in the closed-book format and 11/100 (11%) in the open-book format. They also offered 14/100 (14%) unnecessary PSA tests in the closed-book format and 10/100 (10%) in the open-book format. For follow-up cases, the RAG-LLM tool provided an incorrect recommendation in 1 case: in all 5 instances, it incorrectly recommended a repeat PSA test for a patient with a normal PSA reading. In comparison, junior clinicians ordered 29/120 (24.2%) unnecessary tests in the closed-book format and 23/120 (19.2%) in the open-book format, and missed 24/120 (20%) tests and 13/120 (10.8%) tests in the closed-book and open-book formats, respectively. Overall, the RAG-LLM tool recommended 71 (5 vs 76, 93.4%) fewer unnecessary PSA tests than junior clinicians and missed 59 (5 vs 64, 92.2%) fewer PSA tests that should have been offered.

Results were further analyzed by the following categories of cases: (1) PSA screening recommended; (2) PSA screening not recommended; (3) follow-up of a normal PSA reading; (4) management or follow-up of an elevated PSA reading; and (5) others, including likely spuriously elevated PSA readings from concurrent urinary tract infections, elevated PSA readings in patients with significant comorbidity in whom further or repeat testing would be unlikely to be beneficial, and normal PSA readings in patients with an abnormal digital rectal examination. Results are detailed in Table 1.

**Table 1.** Accuracy and error breakdown of prostate-specific antigen (PSA) testing recommendations by retrieval-augmented generation–large language model (RAG-LLM) and junior clinicians.

Group and category^a^	Unnecessary tests, n (%)			Missed tests, n (%)			Total errors, n (%)	*P* value
	Short interval	Did not require	Subtotal	Long interval	Failed to offer	Subtotal		
Overall (n=220)								
LLM	5 (2.3)	0 (0)	5 (2.3)	0 (0)	5 (2.3)	5 (2.3)	10 (4.5)	—^b^
Human, closed-book	11 (5.0)	32 (14.5)	43 (19.5)	26 (11.8)	14 (6.4)	40 (18.2)	83 (37.7)	<.001
Human, open-book	10 (4.5)	23 (10.5)	33 (15.0)	14 (6.4)	10 (4.5)	24 (10.9)	57 (25.9)	<.001
Category 1: PSA screening recommended (n=55)								
LLM	0 (0)	0 (0)	0 (0)	0 (0)	5 (9.1)	5 (9.1)	5 (9.1)	—
Human, closed-book	0 (0)	0 (0)	0 (0)	3 (0)	10 (18.2)	13 (23.6)	13 (23.6)	.04
Human, open-book	0 (0)	0 (0)	0 (0)	1 (1.8)	10 (18.2)	11 (20)	11 (20)	.11
Category 2: PSA screening not recommended (n=45)								
LLM	0 (0)	0 (0)	0 (0)	0 (0)	0 (0)	0 (0)	0 (0)	—
Human, closed-book	0 (0)	14 (31.1)	14 (31.1)	3 (6.7)	0 (0)	3 (6.7)	17 (37.8)	<.001
Human, open-book	0 (0)	10 (22.2)	10 (22.2)	0 (0)	0 (0)	0 (0)	10 (22.2)	.001
Category 3: normal PSA follow-up (n=45)								
LLM	5 (11.1)	0 (0)	5 (11.1)	0 (0)	0 (0)	0 (0)	5 (11.1)	—
Human, closed-book	8 (17.8)	8 (17.8)	16 (35.6)	0 (0)	3 (6.7)	3 (6.7)	19 (42.2)	.001
Human, open-book	7 (15.6)	7 (15.6)	14 (31.1)	1 (2.2)	0 (0)	1 (2.2)	15 (33.3)	.01
Category 4: elevated PSA (n=40)								
LLM	0 (0)	0 (0)	0 (0)	0 (0)	0 (0)	0 (0)	0 (0)	—
Human, closed-book	0 (0)	0 (0)	0 (0)	20 (50)	1 (2.5)	21 (52.5)	21 (52.5)	<.001
Human, open-book	1 (2.5)	0 (0)	1 (2.5)	12 (30)	0 (0)	12 (30)	13 (28.9)	<.001
Category 5: others (n=35)								
LLM	0 (0)	0 (0)	0 (0)	0 (0)	0 (0)	0 (0)	0 (0)	—
Human, closed-book	3 (8.6)	10 (28.6)	13 (37.1)	0 (0)	0 (0)	0 (0)	13 (37.1)	<.001
Human, open-book	2 (5.7)	6 (17.1)	8 (22.9)	0 (0)	0 (0)	0 (0)	8 (22.9)	.002

a The denominators used for all percentage calculations represent the number of cases in each category multiplied by 5, as each of the 44 case scenarios was independently evaluated by 5 junior clinicians. Accordingly, the overall total is shown as n=220, and the denominators for each category (eg, n=55 for category 1, n=45 for category 2, etc) follow the same calculation method.

b Not available.

Average time taken by clinicians to provide a recommendation was 23 seconds in the closed-book format and 28 seconds in an open-book format. In comparison, the RAG-LLM tool averaged 9.7 seconds per recommendation. Interrater agreements among clinicians for closed-book and open-book responses were Fleiss κ=0.294 (95% CI 0.291‐0.297*; P*<.001) and Fleiss κ=0.321 (95% CI 0.318‐0.324*; P*<.001), respectively, indicating fair agreement. In comparison, Fleiss κ for RAG-LLM tool responses was 1.000 (95% CI 0.998‐1.000; *P*<.001), indicating very good agreement.

## Discussion

### Principal Findings

To our knowledge, this is the first study in the field of urology demonstrating the efficacy of a RAG-LLM tool for clinical decision support. Augmenting LLMs with contextualized information has been shown in other health care domains to reduce instances of hallucination and increase accuracy [14,15]. In this study, guideline-concordant recommendations were made in >95% of scenarios by the RAG-LLM, as compared to the 60%-75% concordance by junior clinicians.

Examining responses that were not guideline concordant, we found that the errors made by the RAG-LLM arose from (1) the rule-based nature of the CCI calculator, which precluded a patient aged 72 years from PSA screening despite strong risk factors for prostate cancer and (2) erroneous interpretation of a normal PSA result as “moderately elevated,” triggering a reactive repeat PSA test, which in actuality was unnecessary. In contrast, the junior clinicians made errors across a broad range of categories, irrespective of seniority or training status.

Analysis of the incorrect recommendation given by the RAG-LLM was undertaken by examining the retrieved guideline chunks and the LLM output for each guideline, followed by the final recommendation. The scenario was that of a 55-year-old man who had been on follow-up for erectile dysfunction, with a PSA screening result of 2.8 ng/mL. The retrieved chunks for both AUA and EAU guidelines contained the information required to answer the clinical scenario.

With regard to the AUA guidelines, the RAG-LLM chain-of-thought process correctly identified an appropriate interval of “regular PSA screening every 2 to 4 years for people aged 50 to 69 years,” but wrongly reasoned that a PSA level of 2.8 ng/mL was elevated and thus recommended a repeat PSA test. As no text in the retrieved chunks suggested the classification of a PSA of 2.8 ng/mL as elevated, we classified this error as a hallucination. Conversely, for the EAU guidelines, contained within the same chunk were the phrases “the most commonly applied threshold for PSA is ≥ 3.0 ng/ml” and “In case of a moderately elevated PSA (up to 10 ng/mL), a repeated test after a few weeks should be considered to confirm the increase.” The RAG-LLM failed to synthesize these 2 pieces of information—specifically, that a “moderately elevated” PSA would range between 3 and 10 ng/mL—and interpreted the PSA of 2.8 ng/mL as moderately elevated. While it recognized the threshold by giving an output stating “given the patient’s age (55 years) and PSA level (2.8 ng/mL), he falls into a category where follow-up intervals of two years may be considered,” it proceeded to reason that “the reference context also suggests that in cases of moderately elevated PSA, a repeated test after a few weeks should be considered,” thus recommending an unnecessary confirmatory repeat PSA test.

In case scenarios where EAU and AUA guidelines provided differing recommendations for PSA testing intervals, the RAG-LLM tool provided both recommendations. In comparison, junior clinicians generally selected a single guideline document as a reference. While not incorrect, their responses were thus qualitatively less comprehensive and thorough than those generated by the LLM tool.

Our study demonstrates that RAG-LLM tools have the potential to augment clinical decision-making by providing guideline-concordant recommendations in real time. While such a clinical task may be relatively simple for an experienced specialist, generalists or junior clinicians may not necessarily have similar familiarity and experience with specialist care. Such clinical decision support tools may prove useful in primary care settings or in care settings where it is practically challenging for a senior clinician to supervise every clinical decision due to time constraints and high patient volume. Patient-specific, guideline-based tools can potentially relieve cognitive burden, shorten learning curves, and improve decision-making time, thus improving overall consistency and efficiency of clinic consultations [16]. Use of RAG-LLM tools as a method to improve guideline adherence can also be a strategy to minimize unnecessary investigations and specialist consultation, thereby reducing costs to patients and public health care systems. In the primary care setting, increased adherence to guidelines has been shown to improve the quality and appropriateness of specialist referrals [17].

From a technical standpoint, RAG-LLM tools are preferable to “off-the-shelf” LLMs. The use of LLMs in clinical medicine engenders concerns of hallucination and resulting inaccurate recommendations, with implications for patient care and safety. Incorporating RAG systems in LLM tools reduces the frequency of hallucinations [18] and is more economical than fine-tuning or pretraining a model from the ground up.

### Limitations

We acknowledge some important limitations to this study, which fall into the clinical and technical domains. First, from a clinical perspective, this study used fictional case scenarios, rather than real clinical cases. While this may limit generalizability and external validation, it is arguably better to perform LLM evaluation on a well-curated set of varied case scenarios, rather than a sample from a general population that would be less likely to feature uncommon or complex cases [19]. This is analogous to the assessment of junior clinicians, where ability would be assessed using a purposefully designed set of cases, rather than a general sample of common cases [20,21]. Future direction includes testing model robustness against retrospective and prospective real-world clinical cases.

A second clinical limitation is the use of the CCI as a tool to estimate 10-year life expectancy. Although the CCI is recommended in the EAU guidelines as a means of estimating life expectancy, it was created in 1987 and has certain limitations in modern practice, such as an incomplete list of comorbidities, assumptions that the effect of comorbidities is additive, and potentially lengthier disease prognoses with modern medical management [22,23]. While comorbidity burden and a patient’s remaining healthy lifespan are key determinants of benefit from any form of screening test, current scoring tools may not adequately capture the nuances of clinical practice and patient assessment and indeed rely on cohort measures of central tendency to estimate life expectancy. We thus envision that such clinical decision support tools would assist clinicians as copilots, maintaining a human-in-the-loop approach rather than functioning as autonomous decision-makers. Additionally, the tool design is modular and separates CCI determination and case analysis into sequential steps, allowing substitution of an alternative comorbidity calculator or omission of this step altogether at the clinician’s discretion.

Third, from a technical perspective, although supplementing LLMs with RAG has been shown to reduce rates of AI hallucinations [18,24], these models are not entirely immune to hallucination. Our RAG-LLM tool provided incorrect recommendations in 1 scenario due to hallucination or faulty reasoning, but erred in a conservative direction, avoiding harms arising from a missed prostate cancer diagnosis. The source of error suggests that current textual documents may require a degree of unwritten human inference, which is not an intrinsic ability that LLMs possess. Identification of these problematic areas in text data and explicit definition of terms may improve reasoning and performance of LLM-based tools. The “black box” nature of many AI or AI-assisted tools [25,26] may present difficulties in pinpointing errors in internal reasoning processes, but use of techniques such as prompt engineering and self-reflective RAG models may help to enhance the accuracy of these models [27]. Variability in performance across different LLMs also needs to be taken into account and balanced against the cost of each model.

### Future Directions

Despite these limitations, RAG-LLM tools retain potential for multiple applications in health care. On the basis of the same system for clinical decision support for guideline-based recommendations, it can also be used retrospectively as an auditing tool to identify areas of guideline discordance in clinical practice [28]. Furthermore, the RAG approach allows future guideline documents to be incorporated much more easily than a fine-tuning or pretraining approach, keeping the tool up-to-date and preventing obsolescence [29]. Prospective real-world model validation based on clinical data, multimodel evaluation, implementation of explainability methods, and expansion of such RAG-LLM pipelines beyond PSA testing to other areas in urology are potential areas for further research.

### Conclusions

In this simulation-based comparative evaluation, we developed a RAG-LLM tool to provide clinical decision support on PSA testing. The tool demonstrated high accuracy, outperforming junior clinicians in making efficient and guideline-concordant decisions. The use of such tools can help increase guideline adherence, improve patient care, and optimize the use of health care resources.

## Supplementary material

10.2196/78393 Multimedia Appendix 1 Supplementary materials, including the design and technical elements of the RAG-LLM tool. LLM: large language model; RAG: retrieval-augmented generation.

## Abbreviations

AI: artificial intelligence
AUA: American Urological Association
CCI: Charlson Comorbidity Index
EAU: European Association of Urology
LLM: large language model
PSA: prostate-specific antigen
RAG: retrieval-augmented generation

## References

[1] Bray F, Laversanne M, Sung H (2024). Global cancer statistics 2022: GLOBOCAN estimates of incidence and mortality worldwide for 36 cancers in 185 countries. CA A Cancer J Clinicians.

[2] Etzioni R, Penson DF, Legler JM (2002). Overdiagnosis due to prostate-specific antigen screening: lessons from U.S. prostate cancer incidence trends. J Natl Cancer Inst.

[3] Pinsky PF, Parnes HL, Andriole G (2014). Mortality and complications after prostate biopsy in the Prostate, Lung, Colorectal and Ovarian cancer screening (PLCO) trial. BJU Int.

[4] Cornford P, van den Bergh RC, Briers E (2024). EAU-EANM-ESTRO-ESUR-ISUP-SIOG guidelines on prostate cancer-2024 update. Part I: screening, diagnosis, and local treatment with curative intent. Eur Urol.

[5] Wei JT, Barocas D, Carlsson S (2023). Early detection of prostate cancer: AUA/SUO guideline part I: prostate cancer screening. J Urol.

[6] Kalavacherla S, Riviere P, Javier-DesLoges J (2023). Low-value prostate-specific antigen screening in older males. JAMA Netw Open.

[7] Clusmann J, Kolbinger FR, Muti HS (2023). The future landscape of large language models in medicine. Commun Med.

[8] Wang D, Liang J, Ye J (2024). Enhancement of the performance of large language models in diabetes education through retrieval-augmented generation: comparative study. J Med Internet Res.

[9] Liu S, McCoy AB, Wright A (2025). Improving large language model applications in biomedicine with retrieval-augmented generation: a systematic review, meta-analysis, and clinical development guidelines. J Am Med Inform Assoc.

[10] Gu Z, Jia W, Piccardi M, Yu P (2025). Empowering large language models for automated clinical assessment with generation-augmented retrieval and hierarchical chain-of-thought. Artif Intell Med.

[11] Charlson ME, Pompei P, Ales KL, MacKenzie CR (1987). A new method of classifying prognostic comorbidity in longitudinal studies: development and validation. J Chronic Dis.

[12] Death and life expectancy. Statistics Singapore.

[13] Wei J, Wang X, Schuurmans D, Koyejo S, Mohamed S, Agarwal A, Belgrave D (2024). NIPS ’22: Proceedings of the 36th International Conference on Neural Information Processing Systems.

[14] Lim DYZ, Tan YB, Koh JTE (2024). ChatGPT on guidelines: providing contextual knowledge to GPT allows it to provide advice on appropriate colonoscopy intervals. J Gastroenterol Hepatol.

[15] Ge J, Sun S, Owens J (2023). Development of a liver disease-specific large language model chat interface using retrieval augmented generation. Gastroenterology.

[16] Chen Z, Liang N, Zhang H (2023). Harnessing the power of clinical decision support systems: challenges and opportunities. Open Heart.

[17] Blank L, Baxter S, Woods HB (2014). Referral interventions from primary to specialist care: a systematic review of international evidence. Br J Gen Pract.

[18] Gilbert S, Kather JN, Hogan A (2024). Augmented non-hallucinating large language models as medical information curators. NPJ Digit Med.

[19] Bai S, Zhang L, Ye Z, Yang D, Wang T, Zhang Y (2023). The benefits of using atypical presentations and rare diseases in problem-based learning in undergraduate medical education. BMC Med Educ.

[20] Surry LT, Torre D, Durning SJ (2017). Exploring examinee behaviours as validity evidence for multiple-choice question examinations. Med Educ.

[21] Ilgen JS, Bowen JL, McIntyre LA (2013). Comparing diagnostic performance and the utility of clinical vignette-based assessment under testing conditions designed to encourage either automatic or analytic thought. Acad Med.

[22] Drosdowsky A, Gough K (2022). The Charlson Comorbidity Index: problems with use in epidemiological research. J Clin Epidemiol.

[23] Charlson ME, Carrozzino D, Guidi J, Patierno C (2022). Charlson comorbidity index: a critical review of clinimetric properties. Psychother Psychosom.

[24] Li H, Huang J, Ji M, Yang Y, An R (2025). Use of retrieval-augmented large language model for COVID-19 fact-checking: development and usability study. J Med Internet Res.

[25] London AJ (2019). Artificial intelligence and black-box medical decisions: accuracy versus explainability. Hastings Cent Rep.

[26] Starke G, Gille F, Termine A (2025). Finding consensus on trust in AI in health care: recommendations from a panel of international experts. J Med Internet Res.

[27] Jeong M, Sohn J, Sung M, Kang J (2024). Improving medical reasoning through retrieval and self-reflection with retrieval-augmented large language models. Bioinformatics.

[28] Goh R, Cook B, Stretton B (2024). Large language models can effectively extract stroke and reperfusion audit data from medical free-text discharge summaries. J Clin Neurosci.

[29] Miao J, Thongprayoon C, Suppadungsuk S, Garcia Valencia OA, Cheungpasitporn W (2024). Integrating retrieval-augmented generation with large language models in nephrology: advancing practical applications. Med Bogota Colomb.

