# Supplementary material for: Performance of Retrieval-Augmented Generation Large Language Models in Guideline-Concordant Prostate-Specific Antigen Testing: Comparative Study With Junior Clinicians
Source: J Med Internet Res. 2025 Nov 19;27:e78393. doi: 10.2196/78393 (PMC12629621; doi:10.2196/78393)
Supplement: Multimedia Appendix 1 [file jmir-v27-e78393-s001.docx]

***Performance of Retrieval-Augmented-Generation large language models in guideline-concordant Prostate Specific Antigen (PSA) testing: A comparative study against junior clinicians***

Joshua Yi Min Tung ^1,2^, Quan Le ^2^, Jinxuan Yao^2^, Yifei Huang^2^, Daniel Yan Zheng Lim^2,3^, Gerald Gui Ren Sng^2,4^, Rachel Shu En Lau^1^, Yu Guang Tan^1^, Kenneth Chen^1^, Kae Jack Tay^1^, Jen Hong Tan^2^, John Shyi-Peng Yuen^1^, Christopher Wai Sam Cheng^1^, Henry Sun Sien Ho^1^

^1^Department of Urology, Singapore General Hospital, Singapore

^2^Data Science and AI Laboratory, Singapore General Hospital, Singapore

^3^Department of Gastroenterology, Singapore General Hospital, Singapore

^4^Department of Endocrinology, Singapore General Hospital, Singapore

**Corresponding Author**

Dr Joshua Tung Yi Min

16 College Road, Block 4 Level 1, Urology Centre, Singapore General Hospital

Singapore 169854

Email: joshua.tung@gmail.com

**Conflicts of Interest**

The authors have no conflicts of interest to declare

Funding: This study was supported by an academic medicine philanthropic fund (the Foo Keong Tatt Professorship in Urology) from the Singapore Health Services Duke-National University of Singapore ("SingHealth Duke-NUS") Joint Office of Academic Medicine

**Keywords**

Artificial intelligence, large language models, retrieval augmented generation

**Word Count**

2,659

**Supplementary Material 1: Charlson Comorbidity Index and Life Expectancy Estimation**

We estimated a patient's life expectancy based on the Charlson Comorbidity Index (CCI) score. This framework takes in a patient's age and the presence of a variety of comorbidities to calculate a score which can be used to estimate the patient's 10-year life expectancy. Refer to Section A1.3 for the variables required to calculate CCI score.

**Information Extraction**

To calculate the CCI score, we prompted LLM to refer to the given patient summary and extract the relevant variables. To improve the reliability of the extraction step, we used language models with function calling capability. We provided the LLM with the schema of the required answer, which contains the variable names, description of the variables and the data types and categories that the variable values can take. Function calling and schema guidance forces outputs to adhere to the desired structure which can be readily processed for downstream tasks. Refer to Appendix 3 for the prompt used to extract patient data and Section A1.4 for the answer output schema used.

First, we sampled 15 test cases from the fictional dataset which had CCI scores manually calculated and annotated by senior clinicians. For each set of LLM output, we computed (1) the number of answers matching human scores exactly and (2) the correlation (Pearson’s-*R*) between human scores and LLM scores. Anthropic Sonnet 3.5 was used as the Large Language Model for information extraction, as it produced the best scores among the tested language models with function calling capability at a lower cost than OpenAI GPT-4 (As of 1-Aug-2024, API price for OpenAI GPT-4 is US$30/1M input tokens & US$60/1M output tokens; API price for Anthropic Sonnet3.5 is US$3/1M input tokens & US$15/1M output tokens).

**CCI Calculation & Life Expectancy Estimation**

The combined CCI score was used to estimate the probability of a minimum 10-year survival based on the following formula[1]:

$$Probability of 10 year survival = 0.983^{exp^{CCI Score x 0.9}}$$

We classified patients expected to live at least 10 years to have a probability of at least **0.5**. This means patients with CCI scores of 5 (probability = 0.214) and above are not expected to have 10-year survival whereas patients with CCI scores of 4 (probability = 0.534) or below are expected to have 10-year survival. We also classified patients 72-years-old or above not to have an expected 10-year survival, based on Singapore’s Male life expectancy at birth of 81[2]. Patients who do not have a 10-year life expectancy are automatically not recommended for PSA screening.

**Supplementary Table 1:** **Schema for information extraction for CCI calculation**

| **Variable Name** | **Type** | **Description** |
| --- | --- | --- |
| age | integer | Age of the patient |
| myocardial_infarction | Enum [YES, NO] | Whether the patient has a history of definite or probable MI (EKG changes and/or enzyme changes). Can be one of [YES, NO] |
| chf | Enum [YES, NO] | Whether the patient has congestive heart failure, exertional or paroxysmal nocturnal dyspnea and has responded to digitalis, diuretics, or afterload reducing agents. Can be one of [YES, NO] |
| peripheral_vascular_disease | Enum [YES, NO] | Whether the patient has intermittent claudication or past bypass for chronic arterial insufficiency, history of gangrene or acute arterial insufficiency, or untreated thoracic or abdominal aneurysm (≥6 cm). Can be one of [YES, NO] |
| cva_or_tia | Enum [YES, NO] | Whether the patient has a history of a cerebrovascular accident with minor or no residual and transient ischemic attacks. Can be one of [YES, NO] |
| dementia | Enum [YES, NO] | Whether the patient has a history of dementia or chronic cognitive deficit. Can be one of [YES, NO] |
| copd | Enum [YES, NO] | Whether the patient has a history of chronic obstructive pulmonary disease. Can be one of [YES, NO] |
| connective_tissue_disease | Enum [YES, NO] | Whether the patient has a history of connective tissue disease. Can be one of [YES, NO] |
| peptic_ulcer_disease | Enum [YES, NO] | Whether the patient has any history of treatment for ulcer disease or history of ulcer bleeding. Can be one of [YES, NO] |
| liver_disease | Enum [NONE, MILD, MODERATE, SEVERE] | Classify if patient liver disease condition, if any. Can be one of [NONE, MILD, MODERATE, SEVERE], whereas SEVERE = cirrhosis and portal hypertension with variceal bleeding history, MODERATE = cirrhosis and portal hypertension but no variceal bleeding history, MILD = chronic hepatitis (or cirrhosis without portal hypertension) |
| diabetes_mellitus | Enum [NONE/DIET_CONTROLLED, MILD, MODERATE/SEVERE] | Whether the patient has a history of diabetes mellitus. Can be one of [NONE/DIET-CONTROLLED, MILD, MODERATE/SEVERE] whereas SEVERE = patients with retinopathy, neuropathy, nephropathy or other end organ damage, MODERATE = patients who had previous hospitalizations for ketoacidosis, hyperosmolar coma, or control and those with juvenile onset or brittle diabetics, MILD = all other diabetes treated with insulin or oral hypoglycemics, but not diet alone, NONE/DIET-CONTROLLED = patients with no diabetes or diabetes can be controlled with diet alone. |
| hemiplegia | Enum [YES, NO] | History of hemiplegia or paraplegia. can of one of [YES, NO] |
| ckd | Enum [YES, NO] | Whether the patient has moderate to severe Chronic Kidney Disease. Can be one of [YES, NO]. For reference, SEVERE = on dialysis, status post kidney transplant, uremia, MODERATE = creatinine >3 mg/dL (0.27 mmol/L) |
| tumor | Enum [NONE, LOCAL, METASTATIC] | Patient solid tumor condition. Can be one of [NONE, LOCAL, METASTATIC] |
| leukemia | Enum [YES, NO] | Whether the patient has leukemia. Can be one of [YES, NO] |
| lymphoma | Enum [YES, NO] | Whether the patient has lymphoma. Can be one of [YES, NO] |
| aids | Enum [YES, NO] | Whether the patient has AIDS. Can be one of [YES, NO] |

**Supplementary Table 2: CCI Scoring for each variable**[1]

| Variable | Variable Description | Scoring Schema |
| --- | --- | --- |
| Age | Patient’s Age | <50: 0 points  50-59: 1 points  60-69: 2 points,  70-79: 3 points  >=80: 4 points |
| Myocardial infarction | History of definite or probable MI (EKG changes and/or enzyme changes) | NO: 0 points  YES: 1 points |
| Congestive heart failure | Exertional or paroxysmal nocturnal dyspnea and has responded to digitalis, diuretics, or afterload reducing agents | NO: 0 points  YES: 1 points |
| Peripheral vascular disease | Intermittent claudication or past bypass for chronic arterial insufficiency, history of gangrene or acute arterial insufficiency, or untreated thoracic or abdominal aneurysm (≥6 cm) | NO: 0 points  YES: 1 points |
| Cerebrovascular accident or transient ischemic attack | History of a cerebrovascular accident with minor or no residua and transient ischemic attacks | NO: 0 points  YES: 1 points |
| Dementia | Chronic cognitive deficit | NO: 0 points  YES: 1 points |
| Chronic obstructive pulmonary disease | Patient’s history of chronic obstructive pulmonary disease | NO: 0 points  YES: 1 points |
| Connective tissue disease | Patient’s history of connective tissue disease | NO: 0 points  YES: 1 points |
| Peptic ulcer disease | Any history of treatment for ulcer disease or history of ulcer bleeding | NO: 0 points  YES: 1 points |
| Liver Disease | Patient’s liver disease status, where  NONE : No liver disease  MILD : chronic hepatitis (or cirrhosis without portal hypertension)  MODERATE: cirrhosis and portal hypertension but no variceal bleeding history  SEVERE: cirrhosis and portal hypertension with variceal bleeding history | NONE: 0 points  MILD: 1 points  MODERATE/SEVERE: 3 points |
| Diabetes Mellitus | Patient’s diabetes status, where  NONE/DIET CONTROLLED: patients with no diabetes or diabetes can be controlled with diet alone  MILD : all other diabetes treated with insulin or oral hypoglycemics, but not diet alone  MODERATE: patients who had previous hospitalizations for ketoacidosis, hyperosmolar coma, or control and those with juvenile onset or brittle diabetics  SEVERE: patients with retinopathy, neuropathy, nephropathy or other end organ damage | NONE/DIET CONTROLLED: 0 points  MILD: 1 points  MODERATE/SEVERE: 2 points |
| Hemiplegia | Patient’s history of Hemiplegia | NO: 0 points  YES: 2 points |
| Moderate to severe chronic kidney disease | Patient’s chronic kidney disease condition  SEVERE: on dialysis, status post kidney transplant, uremia  MODERATE: creatinine >3 mg/dL (0.27 mmol/L) | NO: 0 points  YES: 2 points |
| Solid tumor | Patient’s solid tumor status (NONE, LOCALIZED OR METASTASIS) | NONE: 0 points  LOCALIZED: 2 points  METASTASIS: 6 points |
| Leukemia | Patient’s history of Leukemia | NO: 0 points  YES: 2 points |
| Lymphoma | Patient’s history of Lymphoma | NO: 0 points  YES: 2 points |
| AIDS | Patient’s history of AIDS | NO: 0 points  YES: 6 points |

**Validation of the CCI tool**

We tested several state-of-the-art LLMs which support function-calling. **Appendix Figure A1** shows the performance comparison between different LLMs. OpenAI GPT-4 and Anthropic Sonnet-3.5 had the highest exact match accuracy score (0.81), followed by OpenAI GPT-4o and Anthropic Opus (0.75). In general, the LLM-generated CCI scores had excellent agreement with expert human annotators, except for the Haiku model. For test cases with an inexact match score, LLM-generated scores were usually only a single point apart from the human scores.


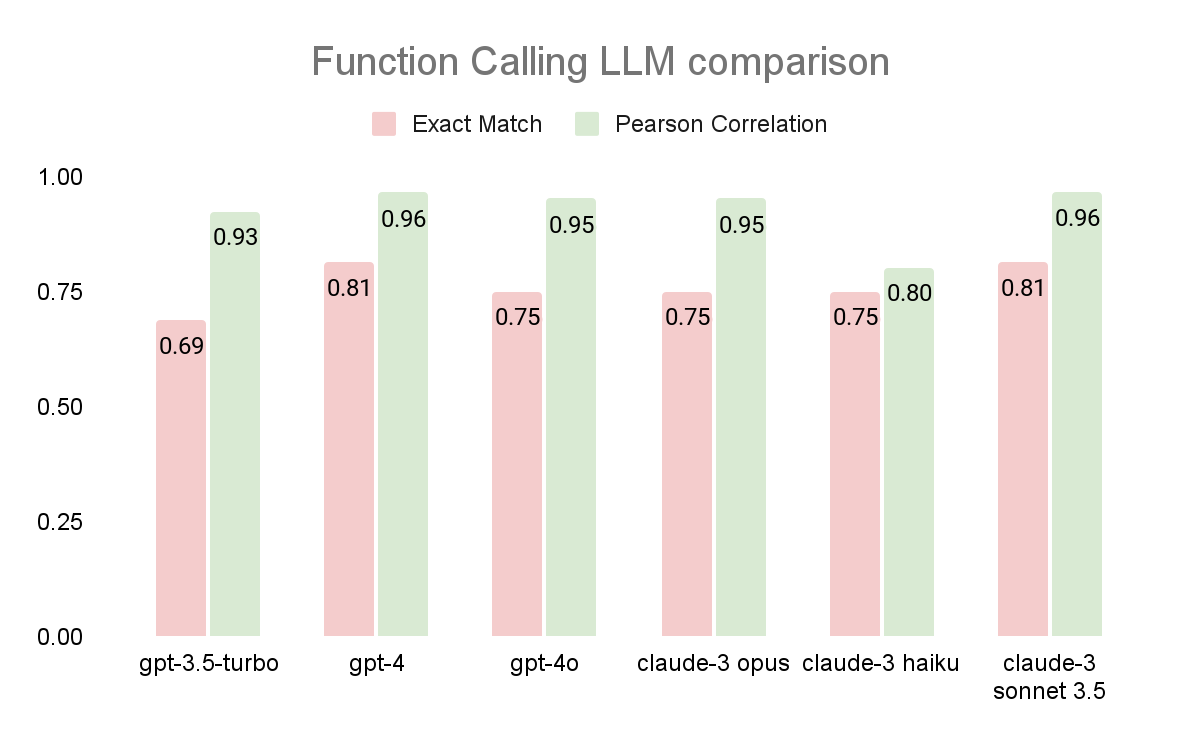


Appendix Figure A1: Performance metrics for CCI calculation between different LLMs

**Supplementary Material 2: RAG System**

**Guidelines Ingestion**


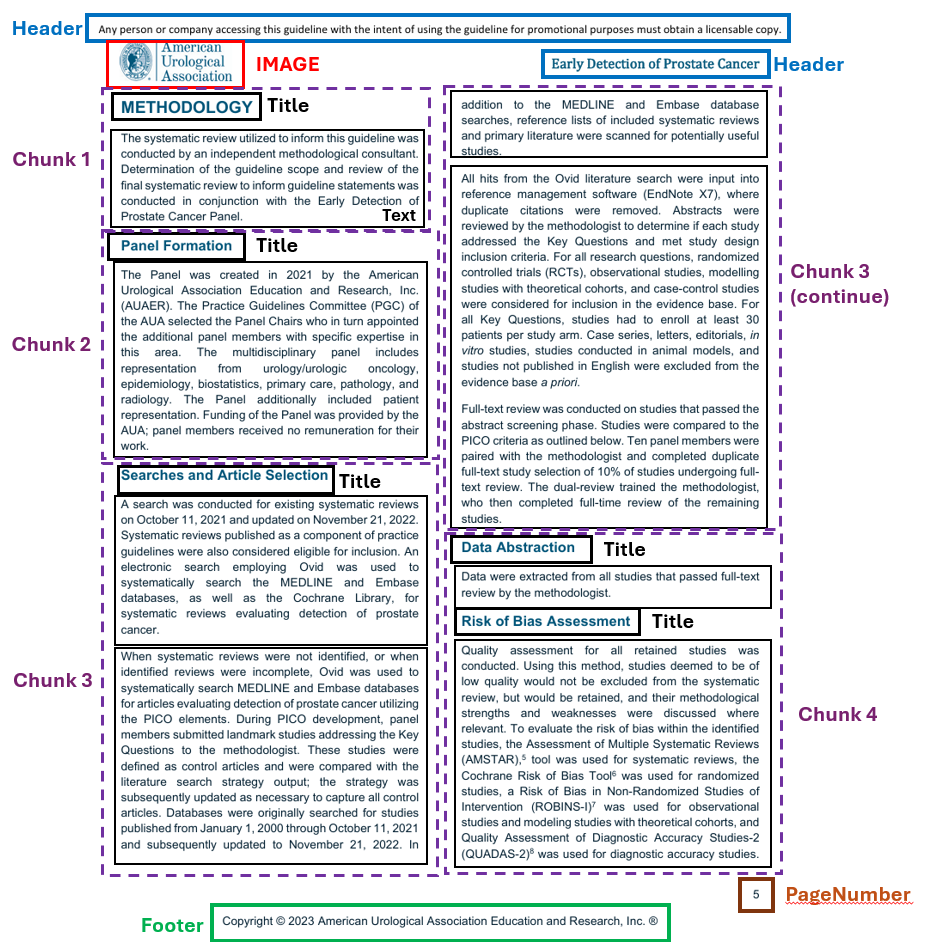


Supplementary Figure 1: Document Chunking Strategy with Unstructured elements

We ingested two guidelines for our knowledge base: (1) Early Detection of Prostate Cancer AUA/SUO Guideline (2023)[4] and (2) EAU - EANM - ESTRO - ESUR - ISUP - SIOG Guidelines on Prostate Cancer[32], both in PDF formats. Both guidelines contain several tables, some of which span across two pages, making it difficult for basic parsing tools such as PyPDF to handle. To overcome this, we used Unstructured (https://unstructured.io/) pipeline to extract different elements (Texts, Headings, Tables, Images) from the original files. Unstructured pipeline consists of a layout extraction step using object detection models, preserving the structure of the extracted contents (including tables and images). Chunks were created by grouping all text elements under it, which ultimately allowed us to maintain the document sections. Refer to Figure A2.1.1 for chunking strategy. Chunks of insufficient length were


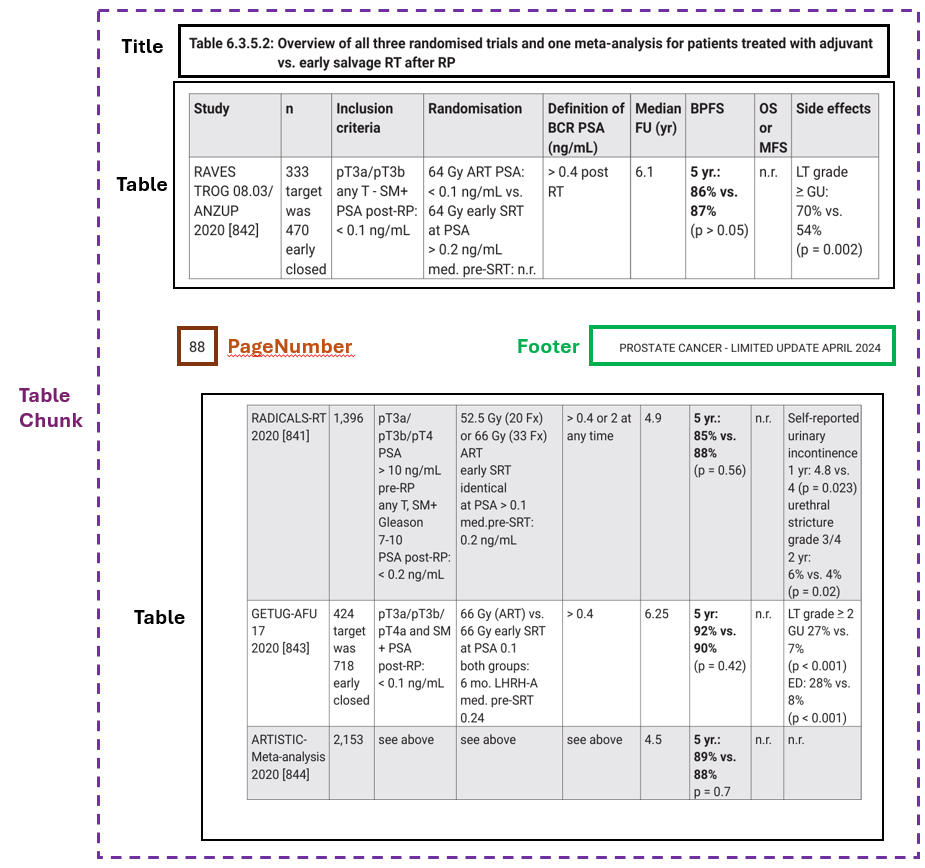


Supplementary Figure 2: Combining Table Elements

merged with the subsequent chunk(s) to ensure that each chunk had a minimum of 256 characters (Chunk 4). Irrelevant elements (Header, Footer, PageNumber, etc) were removed. To address multi-page tables, we validated whether the two subsequent table elements in two consecutive pages formed a valid table (Figure A2.1.2). Finally, pages which only contained irrelevant information (e.g. table of contents, references) were removed.

The distribution of chunk sizes (based on the OpenAI tokenizer) is shown in Figure A2.1.3. Most of the chunks were ~200-300 tokens, with EAU guidelines containing up to more than 800 tokens. For all chunks, we generated embedding vectors using OpenAI text-embedding-3-large model, which represents the semantic representation of the entire text chunks. Finally, all text-vector pairs were indexed inside a vector database, to be used for knowledge retrieval at query time. We constructed one vector database for each guideline.


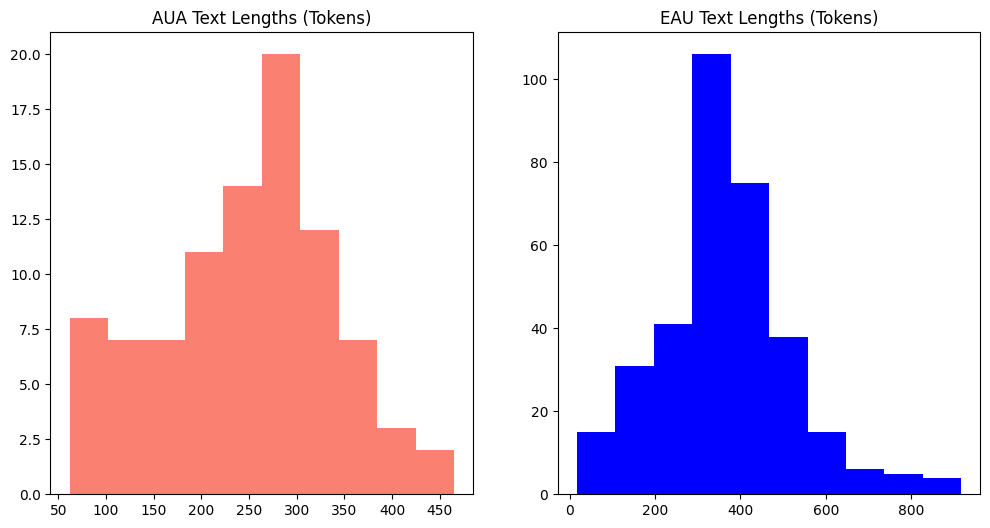


Supplementary Figure 3: Chunk size distribution

**Retrieval Augmented Generation (RAG) Pipeline**

For each database, we created a contextualized RAG pipeline to generate recommendations based on individual patient summaries. At inference time, the retrieval component first searched the vector databases for the information ‘relevant’ to PSA screening with regards to the given individual patient information (e.g. age, history and existing comorbidities, lifestyle habits, etc). The query was mapped to a vector using the same embedding model (text-embedding-3-large), which was then used by the vector databases to search for similar (higher similarity score) guideline information from all contents in the database. In our implementation, we used vector cosine similarity as the similarity metric. For each guideline, we retrieved 5 most relevant guideline texts/tables to the given patient query.

Both the patient query and retrieved contexts were then inserted into a recommendation prompt template to formulate the final prompts. For each patient query, we generated two RAG responses grounded on the two guidelines respectively. We separated the guidelines because the recommendations from different guidelines may not overlap or had the potential to be contradictory, which could confuse the language model during answer generation.

We used OpenAI gpt-4o to generate contextualized answers from each guideline, in order to balance model accuracy with operational costs (API price for OpenAI GPT-4o is US$2.50/1M input tokens & US$10/1M output tokens). To reduce the stochasticity of the model, we set the temperature setting to zero.

**Combining Guidelines**

In the final step of the pipeline, we prompted the LLM tool to combine the answers grounded on both guidelines to generate the final recommendation. In our prompt, we provided instructions tasking the LLM tool to generate answers acceptable by either guideline, and to highlight disagreements between the two guidelines. We also provide 3 examples inside the prompt for better in-context learning. OpenAI gpt-4o with zero temperature was used to combine the answers.

**Supplementary Material 3: Prompts**

**Prompt used for information extraction for CCI calculation**

*SYSTEM: You are a clinician who is an expert in recommendations for PSA screening. Your task is to extract relevant information from a patient profile to calculate Charlson Comorbidity Index for the estimation of life expectancy. You can only extract/make inference to the given PATIENT PROFILE, do not make up new information.*

*Human: Extract information for the following patient:\n{patient_summary}*

**Prompt used for PSA screening recommendation with contextualized guideline information**

*REFERENCE CONTEXT:*

*{context_str}*

*==========*

*You are an expert clinical physician in treatment and management of Prostate Cancer.*

*Your task is to provide recommendations for patients on the screening of Prostate Specific Antigen.*

*Given a PATIENT PROFILE and REFERENCE CONTEXT information extracted from the relevant local guideline, provide recommendations on the next appropriate step on PSA screening, whether it should be performed and if yes, when the PSA screening should be done. Think step by step.*

*Considerations:*

*- Is further investigation required? Does PSA screening need to be repeated prior to further investigation?*

*- Should further screening be recommended or not recommended?*

*- If the patient has prior PSA screening, should PSA screening be repeated? If yes, when is the recommended next screening?*

*- Your response should consider the patient's preferences and his/her functional status.*

*End your response with FINAL RECOMMENDATION of the most appropriate next action, which can be one of:*

*- PSA NOT RECOMMENDED*

*- PROCEED WITH FURTHER INVESTIGATION*

*- PSA RECOMMENDED*

*- PSA REPEATED IMMEDIATELY: PSA should be repeated immediately before further investigation*

*- PSA REPEATED IN X YEARS: Indicate the period until next recommended PSA screening*

*==========*

*PATIENT PROFILE:*

*{query_str}*

**Prompt for combining recommendations**

*You are an expert clinical physician in treatment and management of Prostate Cancer.*

*You are given 2 recommendations based on 2 clinical guidelines. Your task is to combine the 2 recommendations into a single final recommendation.*

*Instructions:*

*- Return a single answer if both recommendations agree.*

*-* *If the period until the next screening differs, give a conservative combined period which covers both recommendations.*

*For example, if AUA recommendation suggests repeated PSA screening after 1-2 years and EAU recommendation suggests repeated PSA screening after 2-4 years, combined answer should be repeated PSA screening after 1-4 years.*

*- If there is fundamental disagreement between the 2 recommendations, state both recommendations and briefly explain the disagreement.*

*=========*

*EXAMPLE 1:*

*- AUA recommendation: PSA screening should be offered*

*- EAU recommendation: PSA screening should be offered*

*FINAL RECOMMENDATION: Both AUA and EAU recommendations suggest PSA screening should be offered*

*EXAMPLE 2:*

*- AUA recommendation: repeat PSA screening after 1-2 years*

*- EAU recommendation: repeat PSA screening after 2-4 years*

*FINAL RECOMMENDATION: repeat PSA screening after 1-4 years*

*EXAMPLE 3:*

*- AUA recommendation: repeat PSA screening after 2-4 years*

*- EAU recommendation: immediate repeat PSA before further investigation*

*FINAL RECOMMENDATION: AUA guideline suggests repeated PSA screening after 2-4 years while EAU guideline to perform repeat PSA prior to further investigation.*

*=========*

*- AUA recommendation: {aua_recommendation}*

*- EAU recommendation: {eau_recommendation}*

**Supplementary Material 4: Sample Case Scenarios**

| **S/N** | **Case** |
| --- | --- |
| 1 | 55 year old Chinese Male, with no significant past medical history or family history of cancer. He had an initial PSA of 4.0 which increased to 8.0 this year. He did not have any recent instrumentation or urinary tract infection. He would like to continue trending his PSA for now before any intervention. When should repeat PSA be done? |
| 2 | 68-year-old male with recent admission to hospital for knee replacement surgery and had acute urinary retention post surgery. He had an indwelling catheter inserted and a PSA was done by orthopedics team which reported to be 10. His prostate volume was 40cc. When should his next PSA be done? |
| 3 | 66-year-old male on finasteride and tamsulosin for LUTS likely secondary to BPH. PSA prior to treatment was 3 and dropped to 1.4 after 6 months of treatment. PSA recently increased to 2.2 recently. |

**References**

1. Charlson ME, Pompei P, Ales KL, MacKenzie CR. A new method of classifying prognostic comorbidity in longitudinal studies: development and validation. J Chronic Dis J Chronic Dis; 1987;40(5). PMID:3558716

2. Death and Life Expectancy. Base. Available from: http://www.singstat.gov.sg/find-data/search-by-theme/population/death-and-life-expectancy/latest-data [accessed Sep 30, 2024]

3. Wei JT, Barocas D, Carlsson S, Coakley F, Eggener S, Etzioni R, Fine SW, Han M, Kim SK, Kirkby E, Konety BR, Miner M, Moses K, Nissenberg MG, Pinto PA, Salami SS, Souter L, Thompson IM, Lin DW. Early Detection of Prostate Cancer: AUA/SUO Guideline Part I: Prostate Cancer Screening. J Urol Wolters KluwerPhiladelphia, PA; 2023; doi: 10.1097/JU.0000000000003491

4. Cornford P, van den Bergh RCN, Briers E, Van den Broeck T, Brunckhorst O, Darraugh J, Eberli D, De Meerleer G, De Santis M, Farolfi A, Gandaglia G, Gillessen S, Grivas N, Henry AM, Lardas M, van Leenders GJLH, Liew M, Linares Espinos E, Oldenburg J, van Oort IM, Oprea-Lager DE, Ploussard G, Roberts MJ, Rouvière O, Schoots IG, Schouten N, Smith EJ, Stranne J, Wiegel T, Willemse P-PM, Tilki D. EAU-EANM-ESTRO-ESUR-ISUP-SIOG Guidelines on Prostate Cancer-2024 Update. Part I: Screening, Diagnosis, and Local Treatment with Curative Intent. Eur Urol 2024 Aug;86(2):148–163. PMID:38614820
